# False Memories for Affective Information in Schizophrenia

**DOI:** 10.3389/fpsyt.2016.00191

**Published:** 2016-11-30

**Authors:** Beth Fairfield, Mario Altamura, Flavia A. Padalino, Angela Balzotti, Alberto Di Domenico, Nicola Mammarella

**Affiliations:** ^1^Department of Psychological Sciences, University of Chieti, Chieti, Italy; ^2^Psychiatry Unit, Department of Clinical and Experimental Medicine, University of Foggia, Foggia, Italy

**Keywords:** false memory, emotion, schizophrenia, scripts, inferences

## Abstract

Studies have shown a direct link between memory for emotionally salient experiences and false memories. In particular, emotionally arousing material of negative and positive valence enhanced reality monitoring compared to neutral material since emotional stimuli can be encoded with more contextual details and thereby facilitate the distinction between presented and imagined stimuli. Individuals with schizophrenia appear to be impaired in both reality monitoring and memory for emotional experiences. However, the relationship between the emotionality of the to-be-remembered material and false memory occurrence has not yet been studied. In this study, 24 patients and 24 healthy adults completed a false memory task with everyday episodes composed of 12 photographs that depicted positive, negative, or neutral outcomes. Results showed how patients with schizophrenia made a higher number of false memories than normal controls (*p* < 0.05) when remembering episodes with positive or negative outcomes. The effect of valence was apparent in the patient group. For example, it did not affect the production causal false memories (*p* > 0.05) resulting from erroneous inferences but did interact with plausible, script consistent errors in patients (i.e., neutral episodes yielded a higher degree of errors than positive and negative episodes). Affective information reduces the probability of generating causal errors in healthy adults but not in patients suggesting that emotional memory impairments may contribute to deficits in reality monitoring in schizophrenia when affective information is involved.

## Introduction

Cognitive deficits are core features of schizophrenia and their role is crucial, particularly in terms of prognosis and functional disability (1, 2). Among these, memory seems to be one of the most impaired cognitive functions, with deficits in semantic and episodic memories (3). Forgetting and memory distortions such as false memories have therefore received much attention (4–7). False memories are memories of an unexperienced event and are rather common in everyday life. Most importantly, false memories have contributed to our understanding of normal memory function, the importance of qualitative features during the encoding and retrieval of complex memories [e.g., Ref. (8)], memory failure in specific brain diseases [e.g., Ref. (9)], and clinically relevant memory distortions in certain patient populations [e.g., Ref. (10)].

### False Memories in Healthy Individuals

Many studies sustain that false memories depend on semantic elaboration since false memories tend to increase as the semantic elaboration of to-be-remembered information increases (11, 12). Moreover, studies on text comprehension have amply demonstrated that when reading stories, people seek to identify factors that link characters and events [e.g., Ref. (13)] in order to achieve coherence. This search for coherence can lead to memory errors, and accordingly, false memories tend to increase as semantic processing, aimed at gaining coherence, increases just as they do when video content is involved (14, 15).

False memories also seem to interact with the affective content of presented material. Numerous studies have demonstrated that production of false memories is modulated by the emotional content of the stimuli [e.g., Ref. (12, 16, 17)] although effects are complex and contradictory. On the one hand, affective content reduces false memories since individuals tend to focus on affective content more than neutral content. This, in turn leads them to encode and remember affective information better. In particular, the decrease in false memories for affective information may be because false memories for emotional stimuli are encoded with more contextual details and thus facilitate the distinction between presented and imagined stimuli (18, 19). In fact, many studies have shown that affective content reduces false memories (18) and leads to monitoring accuracy (12, 20).

On the other, affective content leads to an increase in false memories due to heightened elaboration processes and is further complicated by the fact that positive and negative content also seem to produce different effects on memory (21–25). Indeed, studies on memory recognition have reported an increase in false alarms to emotional stimuli compared to neutral stimuli. These researchers affirmed that affective information induces an attention bias that leads to both enhanced memory accuracy and increased the likelihood of recognizing an emotional stimulus regardless of prior exposure to it (16, 26, 27). Others affirm that false memories for affective content increase since emotions can also serve as organizational factors and can lead to conceptual relatedness. In general, the more stimuli are similar, the more they can be confused in memory. It particular, affective stimuli may also lead to confusion in memory and increase false memories for non-studied affective information since they are conceptually related in that they include emotions (28).

### False Memories in Schizophrenia Patients

More specifically, an increasing number of studies has begun investigating false memories in individuals diagnosed with schizophrenia [for a review, see Ref. (29)]. Results however, are mixed. In general, studies have shown how patients with schizophrenia are more prone to false memories than controls (30). Others, using recognition tasks following the presentation of lists of words phonetically or semantically associated with specific unpresented target words [the Deese–Roediger–McDermott (DRM) task (31)], found no differences in the production of false memories between patients and controls or, in some cases, controls actually made more errors than patients (32–34). Elvevåg et al. (32) found that patients with schizophrenia made more errors (i.e., remember responses) but fewer false recognition errors at recall and argued that the production of false memories in patients may reflect poor memory which makes these population not particularly prone to false recollections. More recently, studies found that patients with schizophrenia are particularly prone to imagination inflation effects. That is, when schizophrenia patients repeatedly imagine an action they often remember the action as carried out (35, 36). According to the Source Monitoring Framework (SMF) (20), these errors occur because imagined events, typically accompanied by more operational attributes, are enriched with so many perceptual details that patients subsequently remember them as having been actually carried out. The same may happen when schizophrenia patients make inferences during story elaboration and lead them to make causal errors. Therefore, it is clear that research on false memories portray a complex pattern of memory errors in schizophrenia, indicating that different factors may differently mediate memory distortions.

Finally, the possible interaction between the affective valence of to-be-remembered material on memory in general, and on false memories in particular, has not been extensively studied in patients with schizophrenia. In particular, studies that adopted recognition tasks have demonstrated that patients with schizophrenia, just as normal individuals, show better performance for negative than positive words (37), while others found that patients remember positive events better than negative ones (38, 39). More recently, using the Remember/Know procedure, a number of studies found that patients with schizophrenia consciously recognized (remember responses) more positive than negative stimuli (40, 41) and recalled more positive than negative autobiographical memories compared to normal controls (42). Less is known, instead, about the interplay between affective information and false memory in schizophrenia.

The present study aims to examine the production of false memories in a group of high-functioning patients with schizophrenia and in a group of healthy adults in order to clarify mechanisms underlying the elaboration of scripted everyday events and the implications of affective content. In fact, the effects of affective content on memory distortions may have critical clinical implications since affective false memories may give rise to psychotic symptoms including hallucinations and delusional thinking (43). Moreover, the elaboration of affective content may also offer insights into aspects of reality monitoring deficits, namely qualitative source encoding errors (e.g., difficulties in encoding source features that typically accompany external vs. internal events) typically shown by patients with schizophrenia.

To this end, we adopted a modified version of the paradigm originally proposed by Hannigan and Reinitz (44) and successively adapted by Toffalini et al. (17) and Mirandola et al. (45) to include affective (both negative and positive) as well as neutral information. This paradigm includes pictorial material representing daily routines designed to investigate two types of memory errors that can occur during script elaboration for coherence and comprehension, plausible, script consistent errors, and causal errors deriving from erroneous inferences.

Based on previous findings (30), we expected patients with schizophrenia to be more susceptible to false memories than healthy controls with both types of errors. With regards to the affective valence of the outcomes on memory, we expected healthy controls, in line with Mirandola et al. (45), to show a general decrease in false alarms when exposed to episodes with affective outcomes compared to those with neutral outcomes. Although we did not make any specific predictions regarding errors in the patient group, given that schizophrenia patients generally do poorly in memory tasks and therefore are less prone to false memories, we may expect fewer errors in general. However, if schizophrenia patients show better memory performance in tasks that include affective information, we may find that false memories increase and be influenced by the affective valence of the outcome.

## Materials and Methods

Twenty-four schizophrenia patients and 24 healthy adults participated in the study. Participants were matched for age, gender, working memory capacity, and mood. Table 1 presents demographic data. Schizophrenia patients were volunteers recruited from the outpatients consecutively attending the community mental health services. None of the patients had been hospitalized within the last 6 months. All patients were able to live in the community with their families or on their own without supervision for major life tasks. The schizophrenia group constitute a “stabilized outpatient” sample, based on their psychological and social functioning as measured by the Global Assessment of Functioning (GAF) Scale with a mean function score of 52.0 (SD = 7.2). All participants in the schizophrenia group used antipsychotic medication with a mean dose in chlorpromazine equivalents of 380 mg/day. Diagnoses were made according to the DSM-IV criteria, as determined by the structured clinical interview for DSM-IV (SCID) (46) by a board certified attending research team of psychiatrists who were trained to a minimum interclass correlation of 0.80. Global psychiatric symptoms in patients were assessed through the Brief Psychiatric Rating Scale [BPRS (47)]. All patients reported normal or corrected-to-normal visual and auditory acuity. Schizophrenia patients and healthy controls had comparable levels of education. All participants completed the forward and backward digit span of the Wechsler Adult Intelligence Scale-Revised [WAIS-R (48)]. The Positive and Negative Affective Schedule [PANAS (49)] was administered to assess current mood. Exclusion criteria for all participants included history of traumatic brain injury, epilepsy, developmental disorder, diagnosable current substance abuse dependence, or other known neurological condition. Written informed consent was obtained from all participants prior to participation. The present study is in accordance with the Helsinki declaration and was approved by the local Institutional Review Board at the University of Chieti.

**Table 1:** **Neuropsychological and demographic characteristics of schizophrenia patients and healthy adults**.

Test variable	Schizophrenia patients (24)		Healthy adults (24)	
	M	SD	M	SD
Age	42.43	6.85	40.63	6.66
Education (in years)	10.5	3.41	11.08	2.78
Digit span forward	4.58	1.31	5.00	1.59
Digit span backward	4.08	1.38	4.88	1.73
PANAS pos	29.05	4.90	31.25	3.23
PANAS neg	18.95	4.25	22.29	6.67
BPRS				
Total	22.56	3.14		
Positive symptoms	7.31	1.67		
Negative symptoms	3.56	1.80		

### Design

Our study adopted a 2(group: healthy adults vs. schizophrenia patients) × 2(type of error: plausible script vs. causal) × 3(valence: positive, negative, and neutral) mixed design.

### Materials

We used nine episodes in our study. Episodes included waking up (i.e., typical morning routine before going to school), going shopping (i.e., young boy going grocery shopping with his mother), dating/meeting a friend (boy and girl meeting in the park), bike trip (i.e., girl going on a bike trip in a downtown area), rock climbing (i.e., boy climbing a wall), track competition (i.e., young girls getting ready for and performing a competition), coming back from a long trip (i.e., girl coming back by train from a trip and entering her home), playing games (i.e., video games in a bar), and a party (i.e., a girl welcoming guests and blowing out candles). Episodes were specifically designed to investigate two types of memory errors that can occur during elaboration for coherence and comprehension, and different affective outcomes (positive, negative, and neutral). Plausible errors (participants say “yes” to an unseen picture that represents a plausible action) are an index that participants actually processed the episodes and causal errors (participants say “yes” to an unseen picture inferred as the cause of the outcome of the episode) are an index of memory distortions determined by the affective outcome of the episode.

In particular, each episode was composed of 15 colored photographs depicting young actors engaged in typical everyday activities. Twelve of the 15 pictures served to create the encoded episode. The remaining three served as plausible errors during the recognition phase. We created two versions of each episode so that targets and plausible script errors were counterbalanced across participants. We also created a single photograph depicting a causal scene that could be inferred as the causal antecedent of the affective outcome for each episode.

**Figure 1:**
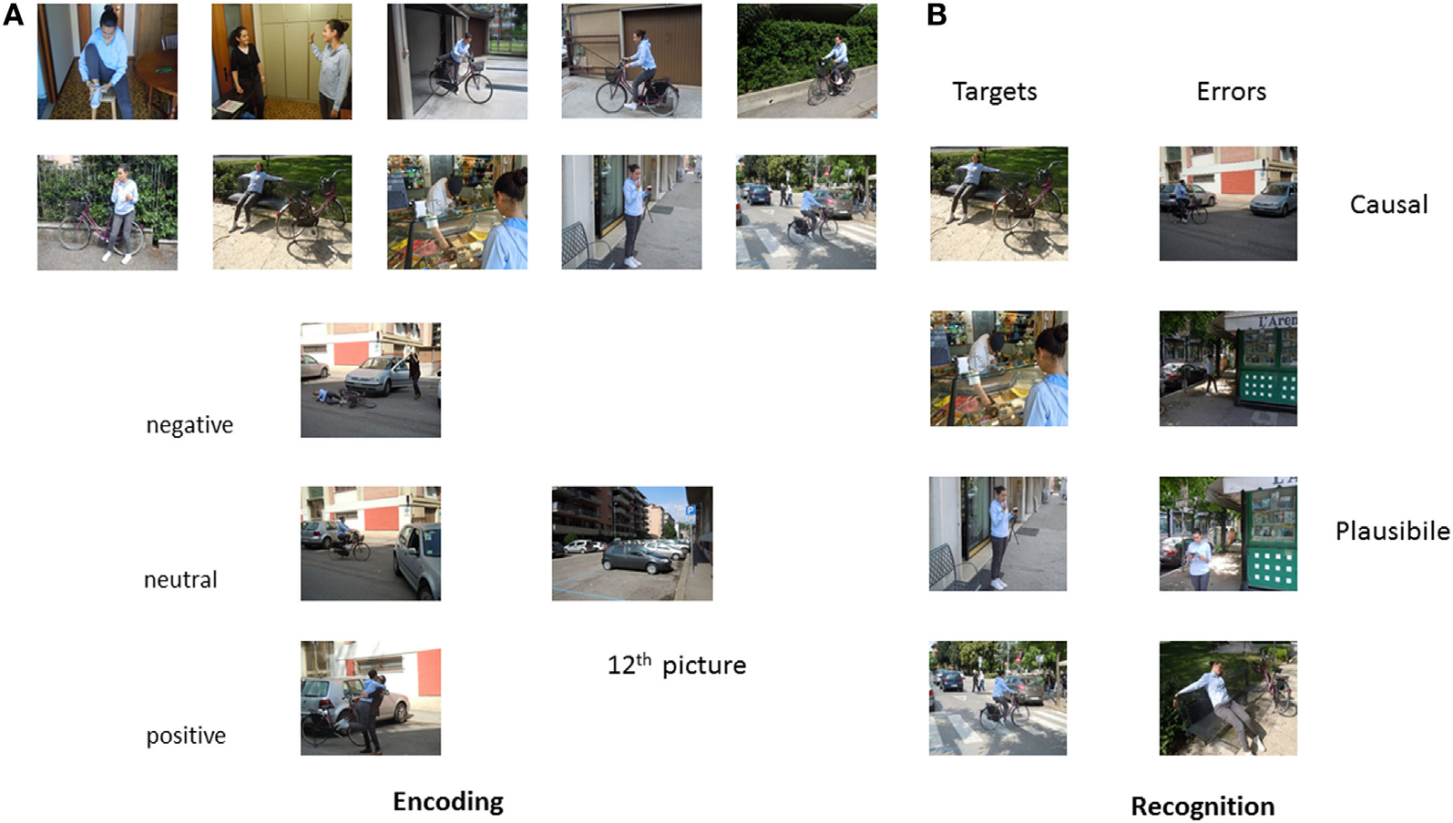
**The bike episode**.

In each episode, the 11th picture communicated the affective outcome of the episode. All of the other photographs were identical across the three affective versions of the episode. For example, the girl and bike on the ground and the driver, out of the car, with her hands on her head (negative), the bike at the entrance with the car already out of the entrance, and the girl getting off the bike (neutral), the girl getting off the bike and hugging a friend (positive) in the “bike” episode. Valence was counterbalanced across participants so that each participant saw three episodes for each affective outcome (Figure 1).

Eighteen independent judges rated valence and arousal levels of the pictures representing the negative, positive, and neutral outcomes using the Self-Assessment Manikin (SAM) scale (50). Paired-samples *t*-tests showed that outcomes significantly differed: negative outcomes had lower valence (M = 2.22, SD = 0.94), *t*(16) = 12.62, *p* < 0.001 and positive outcomes had higher valence (M = 7.67, SD = 0.79), *t*(16) = −15.04, *p* < 0.001, than neutral outcomes (M = 5.04, SD = 0.45). Furthermore, arousal was significantly higher in both negative (M = 7.40, SD = 0.88), *t*(16) = −8.74, *p* < 0.001, and positive outcomes (M = 6.90, SD = 0.93), *t*(16) = −12.92, *p* < 0.001, than in neutral outcomes (M = 3.89, SD = 1.30). Finally, although arousal was slightly higher in the negative than in the positive outcomes, the difference did not reach significance, *t*(16) = 1.64, *p* = 0.12.

Lastly, five unassociated pictures were presented at the beginning and five at the end of the encoding session to avoid primacy and recency effects. Episodes were not introduced by titles and there were no intervals between them.

For recognition, we used a unique sequence of 90 photographs composed of both old and new photographs. In particular, participants saw eight photographs per episode, four old ones from the encoded episode, three new ones that were plausible with the episode, and one never seen causal photograph that could be inferred as a plausible causal antecedent for the outcome of the episode. They also saw 18 single photographs, 9 old and 9 new. Photographs were presented randomly, irrespective of episode, with the constraint that participants never saw two causal photographs in subsequent positions and these were never in the first or last position, or to the slide immediately before the photograph depicting the affective outcome. All actors gave written informed consent to the original authors of the modified paradigm (17, 45) and these authors gave us permission to use the pictorial material.

### Procedure

We tested all participants individually in two sessions. In the first session, we collected demographic information and administered psychological general screening tests. In the second session, participants completed the false memory experimental task. See Figure 1 for a graphical representation of stimuli presentation.

#### Encoding Phase

We told participants that they would see a series of photographs depicting young adults involved in different everyday activities and instructed them to pay close attention to each episode in order to understand what the episode was about for a later memory test. For each of the nine episodes, participants viewed 12 photographs in a logical order; each slide remained on the computer screen for 2 s, followed by a 2-s blank slide. The overall duration of the encoding phase was approximately 9 min.

#### Recognition Phase

Participants complete a self-paced recognition task immediately after the encoding phase. For each image, participants were instructed to respond “yes” if they thought they had seen the image during the encoding phase, and “no” if they thought they had not.

## Results

### Accuracy

A preliminary analysis concerned accuracy. To this end, we calculated two separate accuracy scores, one for unassociated photographs and one for episode photographs. Accuracy scores, in both cases, were calculated as HITs–FA. For episode accuracy scores, false alarms were relative to the episodes regardless of the type of error. We found a significant main effect of group *F*(1,46) = 41.78, *p* < 0.001, η^2^ = 0.48 because schizophrenia patients were less accurate than controls with unassociated photographs (0.54 vs. 0.85). With regards to accuracy for photographs, we found a significant main effect of group *F*(1,46) = 56.05, *p* < 0.001, η^2^ = 0.55 because schizophrenia patients were, in general, less accurate than controls with photographs (−0.05 vs. 0.41). The main effect of valence was also significant *F*(2,92) = 5.79, *p* < 0.01, η^2^ = 0.11. An analysis *post hoc* LSD confirmed that participants were more accurate when the outcome of the episode was positive (0.24) or negative (0.21) with respect to when the outcome was neutral (0.09). There were no differences in accuracy for the episodes with positive or negative outcomes (*p* = 0.44).

### Proportions of False Memories

The principal analysis concerned memory errors, calculated as mean proportions [as done in previous studies, e.g., Ref. (12, 51)], in reporting new pictures as old. Table 2 shows the mean proportions of causal and plausible errors.

**Table 2:** **Mean proportions (with SDs) of false alarms for valence, group, and type of error (plausible vs. causal error)**.

Group	Plausible errors			Causal errors		
	Pos	Neg	Neu	Pos	Neg	Neu
Schizophrenia patients	0.34 (0.04)	0.32 (0.04)	0.51 (0.04)	0.54 (0.08)	0.50 (0.06)	0.53 (0.07)
Healthy adults	0.17 (0.04)	0.19 (0.04)	0.27 (0.04)	0.28 (0.08)	0.43 (0.06)	0.49 (0.07)

**Figure 2:**
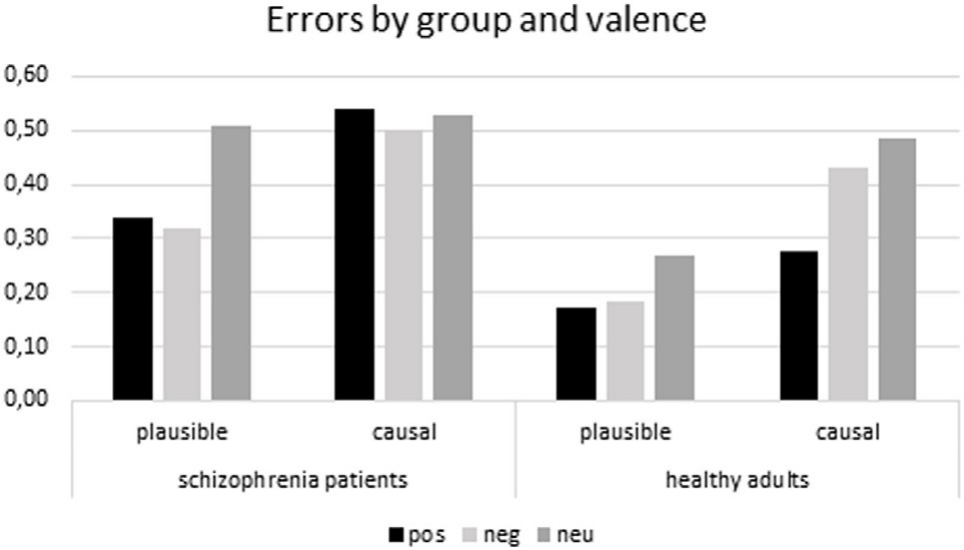
**Mean proportion of false alarms by valence, group, and type of error**.

A 2(group: AD vs. controls) × 2(type of error: plausible vs. causal) × 3(valence of outcome: positive vs. negative vs. neutral) mixed analysis of variance (ANOVA), with the last two factors as within participant factors, on the proportions of false memory as the dependent measure, revealed a significant group effect, *F*(1,46) = 7.17, *p* < 0.05, η^2^ = 0.13 showing that schizophrenia patients made more errors than healthy controls. The main effect of type of error was also significant revealing that plausible errors were more frequent than causal errors, *F*(1,46) = 19.82, *p* < 0.001, η^2^ = 0.30. The main effect of valence of consequence was significant *F*(2,92) = 8.82, *p* < 0.001, η^2^ = 16. *Post hoc* LSD tests revealed how, in general, participants made more errors when the episode contained a neutral outcome compared to a positive (*p* < 0.001) or negative outcome (*p* < 0.01). There were no differences between errors in the negative and positive outcome episodes. The effect was specified by a significant 3-way interaction, *F*(2,92) = 3.20, *p* < 0.05, η^2^ = 0.07. LSD *post hoc* comparisons revealed that neutral plausible errors were more frequent than affective ones in schizophrenia patients (*p* < 0.01) whereas there were no differences between affective and neutral causal errors. Differently, in controls, LSD *post hoc* comparisons revealed that there were no differences between neutral and affective plausible errors while neutral (*p* < 0.001) and negative (*p* < 0.05) causal errors were more frequent than positive ones (Figure 2).

Last, we used Pearson’s correlations, as typically done in studies regarding schizophrenia patients and false memories, in order to exclude possible correlations between false memories, clinical symptoms, and antipsychotic medication (chlorpromazine equivalent) that would confound results. No significant correlations were observed between chlorpromazine equivalent and false memories (plausible and causal errors; all >0.05).

## Discussion

The aim of the present study was to investigate the effect of affective information on false memory in patients with schizophrenia. To our knowledge, this study is one of the first to investigate affective false memories in a group of patients with schizophrenia. The main results of the present study can be summarized as follows. As expected, schizophrenia patients generally made more errors than healthy controls but both groups made fewer errors when episodes contained affective outcomes, consistent with the results of many studies that show how affectively charged material is remembered with fewer distortions than neutral material (18, 25, 45). Moreover, the pattern of false memories as a function of the affective outcome of the episode and type of error differed between patients and controls. Plausible errors in patients with schizophrenia were lower for episodes with affective outcomes compared to neutral ones while they showed a similar pattern of causal errors across all episodes. Differently, healthy controls showed similar patterns of plausible errors across all episodes but a lower number of causal errors for episodes with positive outcomes compared to negative and neutral ones.

Our findings suggest that general emotional enhancement effects in memory may be relatively intact in both healthy adults and patients with schizophrenia. In fact, both groups made fewer errors when the episodes contained affective outcomes compared to neutral ones. One reason for this may be that individuals may more easily reject non-presented information associated with an emotionally relevant episode, due to increased distinctiveness of the scenes presented during the study phase of the experiment (18, 52). Moreover, patients with schizophrenia produced a higher number of plausible and causal errors to episodes than controls. These findings contrast with results from recent studies that found lower levels of both memory accuracy and false memories in schizophrenia patients than normal participants when using non-emotional stimuli and suggest that the production of false memories in patients may not simply reflect poor memory (32, 53). If memory dysfunction in schizophrenia is primarily a reflection of episodic memory deficits, one would expect a higher number of false memories as memory accuracy for presented information increases. However, in the present study, we found a lower number of false memories (plausible and casual errors), despite poor recognition in the patient group. Thus, our results cannot be explained as a reflection of a relative increase in memory accuracy in patients with schizophrenia.

In addition, most of the research that has investigated schizophrenic patients’ susceptibility to false memories used non-emotional words or sentences as stimuli. It is, therefore, conceivable that apparent differences between our results and those from previous studies may reflect differences in the stimuli used. Our results are in line with existing evidence suggesting that affective information may both *heighten* memory accuracy and, in some circumstances, favor the endorsement of non-experienced events (16, 26). These findings suggest that the higher number of causal errors in patients may be partially due to a fundamental deficit in executive processes (i.e., breakdown in attentional–inhibitory control). Recent studies have suggested that an interaction between frontal executive cognitive control circuits and limbic processing systems sub-serves a critical function in the regulation of attention in the presence of emotionally arousing information. For instance, stimuli that engage frontal executive cognitive control circuits simultaneously induce deactivation or suppression of limbic cortical regions (54, 55). Increasing evidence indicates that individuals with schizophrenia may engage such inhibitory processes for shorter durations, resulting in poor frontal control when elaborating affective stimuli (56). Patients with executive dysfunctions in our study may fail to suppress highly activated episode relevant pictures (e.g., pictures of events that typically occur in a given type of episode) and inferences and consequently incorporate these pictures when remembering.

Another way to account for the higher number of causal errors observed in the group of patients refers to the SMF (20). According to the SMF, memory for specific perceptual attributes experienced during an event indicates something actually seen or encoded. Conversely, the lack of perceptual details may indicate that an item was generated internally. Source decision then, can be made by considering the absence of memory for certain details according to an if–then strategy (i.e., if I would have seen it I would have remembered it). Interestingly, when an imagined event is rich in sensory and contextual information (qualitative features that typically belong to external events), participants may believe that the event really happened (57). Here, the higher number of causal errors in patients compared to controls indicates that patients make inferences as they experience emotional events. Subsequently, these inferences might be misremembered as previously experienced components of the encoded episode, probably because the imagined events were so rich in sensory and contextual information and virtually undistinguishable from those scenes presented during study. This study complements a previous study carried out in our lab that showed that the susceptibility to false memories might be a reflection of imagination inflation effects in patients with schizophrenia (35). In this latter study, participants imagined the event so vividly that they were led to believe that the event had truly occurred, although it did not suggest that patients may have difficulties in modulating overlearned behaviors. Interestingly, it has been suggested that “over mentalizing,” that is over generating hypothesis about others’ mental life may represent a specific deficit in schizophrenic patients with positive symptoms (58). It is possible that these individuals may have difficulty controlling highly activated interpretations by over attributing mental states and, thus, produce a higher number of false memories. Further studies are needed to conclude a casual association between this Theory of Mind error type and the development of false memories.

It is also possible that patients may have inferred source information based on snap judgments (59). If so, the higher number of inferential errors in patients may indicate that patients jump to conclusions and often rely on partial information when accepting an item as having been studied compared to controls (60).

Concerning the influence of affective valence on memory performance, we observed a lower number of causal errors in controls when episodes contained a positive outcome compared to a negative one, whereas patients with schizophrenia exhibited similar number of causal errors in both positive and negative episodes. The memory advantage for episodes with positive outcomes in controls replicates previous findings from studies conducted on normal participants showing that positive events may protect individuals from making false memories (38, 39). Previous studies conducted on patients with schizophrenia found that patients recognized more positive than negative pictures, and recalled more positive than negative autobiographical memories than controls (37, 42). However, our results are consistent with another study conducted by Kayton and Koh (61) in which, contrary to controls, patients recalled unpleasant words and pleasant words equally well, indicating an emotionally undifferentiated recall. It is possible that the patients in our study had trouble remembering specific perceptual characteristics of stimuli due to executive deficits and accordingly showed a higher number of false memories for both negative and positive emotional events. Recently, a number of studies have investigated the effects of psychostimulant and psychotropic drugs on true and false memories in healthy volunteers (62). Although the results in those studies are mixed and inconsistent, there is some suggestion that false memories are more susceptible to drug effects than the true ones (63). This information is clinically important for patients with schizophrenia who use psychotropic medications. Particularly, Guarnieri et al. (64) found that sulpiride, a selective dopamine D2 antagonist, did not affect remembered stimuli (emotional and non-emotional) but heightened false recognition only for emotionally charged items (positive and negative) and not neutral ones. Therefore, it could be argued that, at least in part, the higher number of false memories in our patients might be related to the effects of antipsychotic drugs. However, this is not the case. If the higher number of false memories for emotionally charged items is primarily a reflection of the effects of psychotropic medication, one might expect more causal errors for episodes with affective outcomes compared to neutral ones, while our patients showed a similar pattern of causal errors across all episodes. Moreover, we did not find significant correlations between chlorpromazine equivalent and emotional false memories.

Finally, the current study has some limitations. In the present study, we did not use the Remember/Know procedure. Previous research investigating the different states of awareness accompanying inferred-based memory errors provided evidence that the underlying memory processes that give rise to plausible errors are fundamentally different from those that give rise to errors based on causal inference. Specifically, plausible errors may be based on a feeling of familiarity in the absence of explicit recognition and may result from the activation of high-schema-relevant items (e.g., events that typically occur in a given type of episode). Causal errors, instead, result from specific inferential processes [e.g., diagnostic processes that function to attribute effects to likely causes (65, 66)]. In the light of this, our findings are contrary to those found in a series of studies that investigated the influence of emotion on autonoetic awareness in schizophrenia. Indeed, these studies found no difference between remember and know responses when affective stimuli were involved (40, 41). Future studies need to adopt this type of procedure to investigate whether patients with schizophrenia show the same pattern of performance when using complex information such as pictorial episodes.

In sum, in this study we found that schizophrenia patients make a higher number of false memories when episodes lack affective information, especially for new plausible information. Further, in terms of causal false memories, affective information reduces the probability of generating causal errors in healthy adults but not in patients suggesting that emotional memory impairments may contribute to deficits in reality monitoring in schizophrenia when emotional information is involved.

We interpret this finding as reflecting a breakdown in attentional–inhibitory control due to decreased efficiency in cognitive control processes mediated by frontal systems, reported previously in schizophrenia. Our results suggest that individuals with schizophrenia may experience difficulty differentiating between the effects of valence, which determine the salience of an event and, consequently, patients may be more likely to erroneously recognize previous events when these events have emotional content.

## Author Contributions

BF and NM developed the study concept. All the authors contributed to the study design. Testing and data collection were performed by FP, AD, and AB. BF and AD performed the data analysis and interpretation under the supervision of NM and MA. BF drafted the manuscript, and MA and NM provided critical revisions. All the authors approved the final version of the manuscript for submission.

## Conflict of Interest Statement

The authors declare that the research was conducted in the absence of any commercial or financial relationships that could be construed as a potential conflict of interest.
